# Calcination of Clay Raw Materials in a Fluidized Bed

**DOI:** 10.3390/ma14143989

**Published:** 2021-07-16

**Authors:** Katarzyna Kaczyńska, Konrad Kaczyński, Piotr Pełka

**Affiliations:** Faculty of Mechanical Engineering and Computer Science, Institute of Thermal Machinery, Czestochowa University of Technology, al. Armii Krajowej 21, 42-201 Czestochowa, Poland; konrad.kaczynski@pcz.pl (K.K.); piotr.pelka@pcz.pl (P.P.)

**Keywords:** clay raw materials, fluidization, calcination, thermal enrichment

## Abstract

Clay raw materials are diverse in terms of their mineral composition, as well as the content of colouring oxides and their physical properties. Determining the suitability of raw materials for various purposes requires comprehensive studies on their properties, as well as their appropriate correction, which is possible through the use of appropriate modification techniques. One of the most commonly used technologies for the enrichment of clay raw materials is to subject them to high temperatures, which, depending on the temperature regime used in the technological process, may cause the decomposition and removal of some addditional components (e.g., carbonates), as well as the removal of water and dehydroxylation of clay minerals, reversible structural changes, and the complete and permanent reconstruction of the mineral phases. This paper presents a new application for fluidization technology in the calcination of clay raw materials. The results of the experiment show that the fluidization method is competitive compared to the technologies that have been used so far, as a result of, inter alia, the much shorter time period required to carry out the calcination process and, consequently, the much lower energy expenditure, the high efficiency of burning coal, and the lower CO_2_ emissions resulting from the mixing taking place in the reactor.

## 1. Introduction

The exploitation and enrichment of lignite is inextricably linked with the problem of accompanying minerals, including clay minerals [1,2,3]. Clay rocks have long attracted interest as raw materials. The first research and publications on the use of minerals accompanying lignite deposits originated in the late 1940s. During the next several dozen years, growing interest in them became noticeable, often ending with the documentation of deposits and raw materials [2]. This, in turn, made it possible to start exploitation, and conditioned their industrial use. The use of accompanying minerals should be considered both in economic terms, with respect to the possibility of practically managing these minerals, thus creating an opportunity to increase the resource base, as well as in terms of environmental protection, by avoiding the massive accumulation of resources, which contributes to the deterioration of the environment and the waste of acquired raw materials [4]. Clay materials accompanying deposits of lignite may be availed of in the ceramics industry, as well as in rubber, polymers, and fibreglass (at fractions of up to 15 micrometres), whereas larger fractions may be used in construction ceramics, ceramic stoneware, the production of white cement and fireproof materials, in drilling (mud), hydroinsulation of landfill sites, hydroinsulation of rock mass, and recultivation of sandy soil, as well as adsorbents of organic pollution and water treatment [1,5].

The process of clay resource management shows that the most economical way to use them is their enrichment and processing. Most often, the technological processes of processing clay raw materials are associated with changing their properties at high temperatures. Depending on the purpose of the thermal transformations and the expected quality parameters of the obtained products, the range and scope of the temperatures used may vary. Calcination is a type of roasting that involves heating a chemical compound to a temperature below its melting point. It is a process of the thermal decomposition of minerals resulting in the release of gas, e.g., CO_2_ (carbonate minerals) or H_2_O (clay minerals, gypsum). This process is most often carried out in furnaces or reactors in controlled atmospheres at temperatures in a range of 550–1150 °C. The increase in temperature affecting the clay raw materials causes the following reactions to occur: dehydration, dehydroxylation, and the synthesis of new crystalline phases within the formed forms [6,7,8,9]. In the process of drainage, there is a loss of water and the combustion of organic substances, in which the process deviates from the ambient temperature by up to 200 °C. In the range of temperatures between 200 and 400 °C, the process of decomposition of gibbsite occurs [6]. Kaolinite dehydroxylation begins at temperatures above 400 °C [10], with its maximum being recorded on basis of curves of thermal differential analysis at about 506 °C. As a result of dehydroxylation, an amorphous X-ray anhydride is formed, namely, metakaolinite, which is commonly used as a pozzolanic additive in mortar and concrete and which enables the improvement of the mechanical parameters and durability of cement composites [11,12,13]. Metakaolinite is a fine-grained material, and 99.9% of its particles are smaller than 16 μm, with an average size of 3 μm. The main components of metakaolinite are silicon dioxide (SiO_2_) and aluminium oxide (Al_2_O_3_). The remaining oxides constitute a small share in the total mass. A proportion of 12% of kaolinite OH groups are preserved in the structure of metakaolinite. Rashad, in [14], compared the times and temperatures of roasting given by many researchers. Most of them indicated an optimal range of roasting temperatures at a level of 650–850 °C. The roasting temperature influences the pozzolanic activity of the metakaolin formed. As metakaolinite is heated further, OH groups are gradually eliminated, and its structure is partially destroyed, producing an aluminosilicate spinel phase and silica. Under the influence of temperatures exceeding 900 °C, metakaolinite is sintered and mullite is formed, which is an inert component [15]. In the calcination process, the main element of the installation is a furnace in which the clay raw materials are subjected to high temperatures, thanks to which the products acquire the required properties. The most commonly used so far have been rotary and shaft furnaces, although currently, shaft furnace technology is being abandoned. In turn, the fluidization method has many advantages over rotary kiln technology, e.g., a much shorter time required for carrying out the calcination process, and high firing efficiency due to the mixing taking place in the reactor, which has resulted in its increasingly wide use [16,17,18]. The novel innovation here is that of the installation of instant calcination, in which the process of calcination lasts several seconds (0.5–12 s). The advantage of this method is the high level of energy efficiency due to the possibility of the execution of several cycles of heat recovery, as well as the high level of pozzolanic activity of the metakaolinite, although in this method drying is required, as well as grinding of the primary clay material into powder [6]. In [19], fluidized bed roasting was used to extract vanadium from hard coal, while in [20], the fluidized bed technology was used to recover low-quality hematite during magnetizing roasting. On the other hand, Shuai [21] investigated the effect of the calcination temperature on the activation behaviour of kaolin by calcination in a fluidized bed. The indicators for the evaluation of calcined products were as follows: weight loss rate, whiteness, chemical oxygen demand and dissolution degree of aluminium. Moreover, the thermal behaviour and the reaction mechanism were discussed on the basis of TG-DSC, XRD, FT-IR, PSD and SEM. The raw kaolin used in this study was collected from Shuozhou, China. The fluidized bed calcination experiments were performed in a custom lab-scale fluidized bed. During the experiment, 15 g samples of raw material were fed into the reactor. The sample underwent dry grinding to 15 µm, occupying 85% of the specific surface area of 1.99 m^2^/g, with a minimum fluidization velocity of 0.07 m/s. The calcination lasted 180 min. The fluidized bed reactor was cooled quickly to room temperature with nitrogen and the samples were taken out for analysis. They proved that fluidized bed calcination was an efficient technology for thermal activation with the aim of obtaining calcined kaolin with excellent properties in an ideal temperature field [21].

However, in [22], the use of various unconventional sources of energy was proposed, such as concentrated solar power (CSP), which may ensure the high temperatures necessary in the traditional process of calcination.

This paper presents a detailed analysis of the possibility of thermally processing various clay raw materials (chemical composition, graining) in a fluidized bed. The special advantage of the presented technology is the possibility of the reactor’s continuous operation and the short residence time of the material in the bed, compared to the technologies mentioned above [21], lasting less than several dozen minutes.

The aim of this paper was the detailed analysis of the thermal possibilities of alterations in the fluidized layers of the chosen raw clay materials derived from Polish deposits.

## 2. Research Methodology

In the first stage of the research, analysis and preliminary experimental tests were carried out regarding the possibility of calcining the clay material in the fluidized bed, depending on the process parameters and material properties of the charge, including:

- Technical analysis of clay materials;
- Analysis of the particle size distribution of clay materials;
- Determination of the velocity of fluidization;
- Preliminary experimental tests at an ambient temperature;
- Preliminary tests of the calcination process of raw clay material at the temperature of 850 °C in a fluidized bed.

Subsequently, experimental tests were carried out in a fluidized bed reactor and tests were carried out to verify the calcination process, i.e.:

- The degree of calcination was determined;
- Particle distribution was compared before and after the calcination process.

The research material constituted granulated enriched silt from Polish reserves accompanying lignite that was acquired in the process of water purification and then enriched in an intricate manner using high-alumina kaolin clay accompanying the deposits of vein quartz. The kaolin materials from this source in this process achieved the enrichment of silt by means of filtration, thanks to which enrichment becomes possible.

### 2.1. Granulation Process of Enriched Clay Material

The granules were prepared in order to fluidize the enriched raw material. The granulation process requires an enriched raw material that has a standardized moisture content within a narrow range of 16 ± 0.5%. To ensure such humidity, the filter cakes of the enriched raw material were conditioned in atmospheric conditions, and after obtaining the required humidity, they were preliminarily shredded in a semi-technical hammer mill with a diameter of 800 mm, equipped with an evacuation partition of 16 mm. The pre-comminuted raw material was then granulated on a grinding sieve granulator. Woven screens with mesh sizes of 3.4 and 6 mm and a perforated screen with round holes with a diameter of 6 mm were used in the tests to obtain granules. The granulate obtained from the conveyor was spread into particle classes on a vibrating sieve.

The particle classes were sown as follows:

- Granules of 0.5–5 mm when rubbed through a 6.0 mm sieve;
- Granules of 0.5–3 mm when rubbed through a 6.0 mm sieve;
- Granules ≤1 mm when rubbing a 1.0 mm through a 3.0 mm sieve;
- Granules of 1.0–3.0 mm when rubbed through a 4.0 mm sieve;
- Granules ≤3 mm when rubbing a 3.0 mm sieve through a 4.0 mm sieve.

The undersized and oversized granules obtained during the process of spreading the granules were moistened, mixed to a state of homogenization, and returned to crushing, so that all the raw material to be granulated was finally obtained in the form of granules with complex particle sizes. The granules of the required particle class were dried on a screw dryer with a heated screw tube, which was especially designed for the purpose of carrying out the drying process under the conditions of screw transport. Under the conditions of transport by the dryer screw, the granulate took on a spherical shape; this was especially for the first passage through the dryer screw, when the moisture of the granulate was the highest. The required drying of the granulate, to a moisture content of below 5%, was obtained after passing the granulate 4 or 5 times through the dryer.

### 2.2. Characteristics of the Research Material

This research work concerned 4 groups of materials, differing in terms of particle size. After chemical, technical and particle analyses, and after determining the fluidization parameters, some of them were selected for experimental tests.

Types of materials tested:

1. A—kaolin industrially enriched at the division boundary of 100 μm. The useful ingredient has a high Al_2_O_3_ content (about 40%):
  - Granules < 2 mm
2. B—clay raw material comprehensively enriched with high-alumina kaolin accompanying deposits of vein quartz. In the enriched product, the mass ratio of substrates is 1:1. The enrichment aims to the Al_2_O_3_ content and reduce the Fe_2_O_3_ content:
  - Granules < 5 mm;
  - Granules < 3 mm;
  - Granules < 1 mm;
  - About 1–3 mm granules;
3. C—material sample with a very high degree of carbonization, laboratory enriched at a division limit of 100 μm:
  - Granules <2 mm.
4. D—clay raw material aerated and carburized with coal suspension, stabilized with bone glue, electrokinetically dehydrated, sieve granulated:
  - Granules 1–4 mm;
  - Granules <1 mm.

Research has been carried out on the chemical composition of silt when the material was deprived of transient moisture, as well as crystal-bound water [23]. Table 1 presents the results of the chemical analysis of the clay materials. Sample D consists of approximately 80% of sample B, approximately 1% bone glue and approximately 20% of lignite dust.

### 2.3. Results of Technical Analysis of Clay Raw Materials

Technical analysis of the clay raw materials was carried out in accordance with Polish standards applicable to solid fuels. The determination of the moisture content was conducted by the drying method from the analytical sample in accordance with the determination of moisture content PG-G-04511:1980—solid fuel. The determination of the volatile content was conducted in accordance with the standard PN-81/G-04516. The determination of the content of clay materials was conducted by the method of slow incineration in accordance with the standard PN-80/G-04512. During the technical analysis an electric drier was used, as well as a muffle furnace and laboratory scales with a precision of 0.001 g. Each determination was conducted a minimum of three times.

Table 2 contains the results of the technical analysis of the clay raw materials. The raw material with the largest particle size (5 mm) also possessed the highest moisture content, while the lowest moisture content was shown by material B, with particles size below 3 mm. The clay materials after the carburizing process had a volatile matter content in the range of 18–19.5%.

### 2.4. Results of Particle Analysis of Clay Materials and Determination of Fluidization Parameters

The particle analysis was performed using an automatic particle size analyser AWK Drop Particle Analyzer. On the basis of the measured values, the parameters and particle size distributions of the material were calculated, specifically defining the analysed set of particles. The analyses were performed in triplicate for each material. Table 3 shows the average results of the analyses.

**Figure 1:**
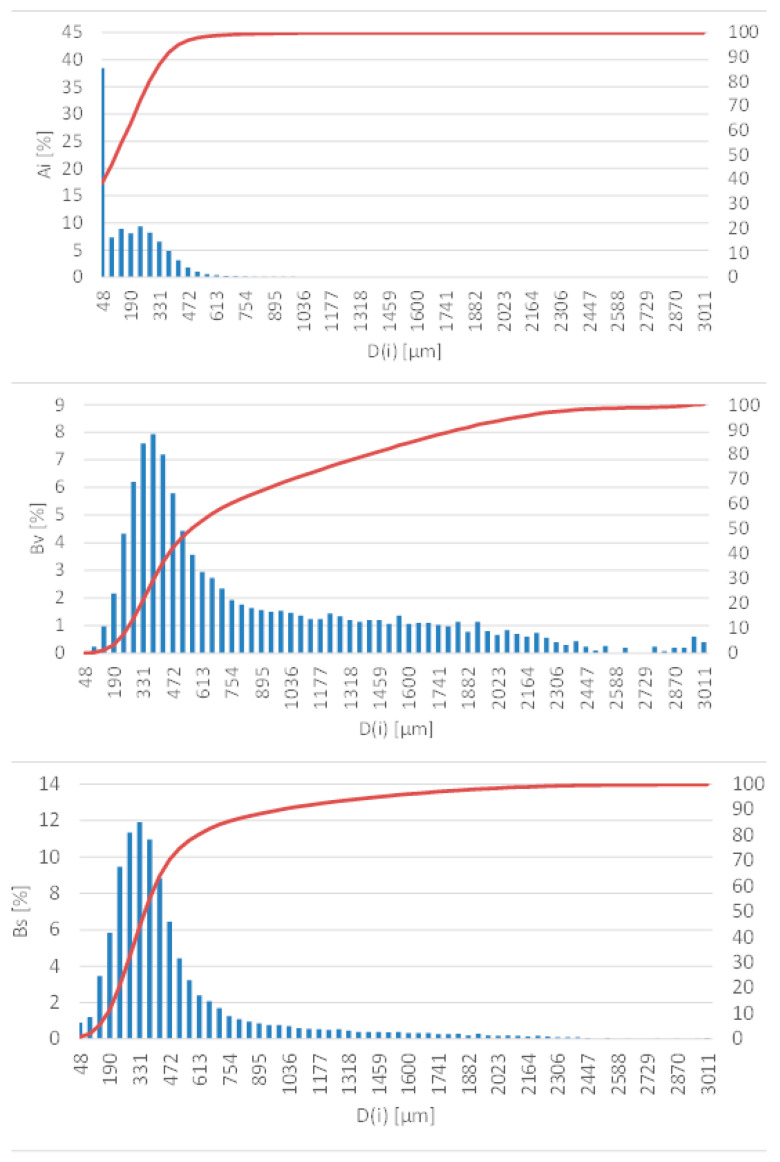
Quantitative (Ai), volumetric (Bv) and area distribution (Bs), A < 2 mm.

Figure 1 presents the average quantitative, volumetric and surface distribution (minimum of three measurements) of A, with a granule size <2 mm. The curved line marked in red colour represents the sum of the fraction. Figures presenting the average quantitative, volumetric and surface distribution (minimum of three measurements) for all tested samples can be found in the Supplementary Materials.

### 2.5. Determination of Fluidization Parameters

Based on the particle analysis of the tested clays, the equivalent diameter of the so-called Sauter diameter (d_a_) was established. On its basis, the Archimedes number (Ar) was determined, then the Reynolds number (Re), the minimum velocity of fluidization (U_mf_), the terminal velocity (U_t_) and the velocity of pneumatic transport (U_tr_) [24]. Based on the particle size distribution of the tested materials and their mass density, the tested materials were classified on the basis of their classes of loose materials in accordance with the Geldart classification [25]. Most of the materials were classified into group B, and on this basis it was concluded that the minimum velocity of fluidization was equal to the minimum velocity of the bubble layer (U_mb_). The clay raw material B, with a granule size <5 mm, was an exception, as its equivalent diameter caused this material to be classified into group D—prone to fountaining—while materials with the finest particles, below 1 mm, were classified into group C—prone to sticking, difficult to fluidize. Table 4 presents the calculation results of the fluidization parameters for the analysed samples for two temperatures: ambient and 850 °C. In the case of samples with an equivalent diameter below 0.5 mm, the actual material density was assumed to be 2500 kg/m^3^; for materials with a larger particle size, an apparent material density of 650 kg/m^3^ was assumed in the calculations due to the high porosity of large particles of the material.

## 3. Preliminary Experimental Studies at Ambient Temperature

**Figure 2:**
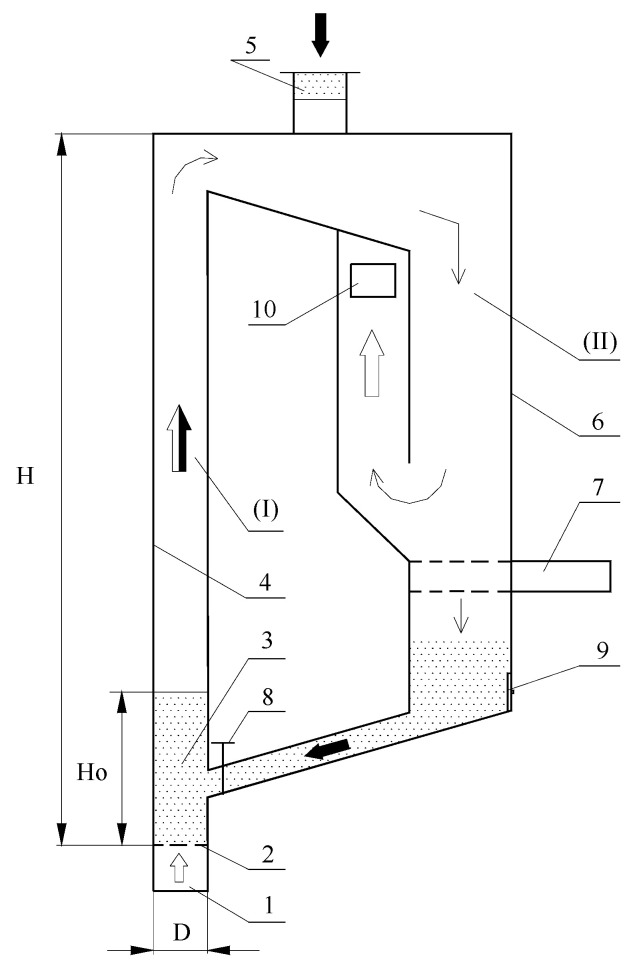
Detailed diagram of the fluidization column (side view): 1—air space, 2—grate, 3—layer of inert material with a height Ho, 4—front glass of the fluidizing column, 5—material supply point, 6—separator rear window, 7—drawer for measuring the amount of circulating material, 8—control valve, 9—material discharge, 10—air outlet for non-separated material.

**Figure 3:**
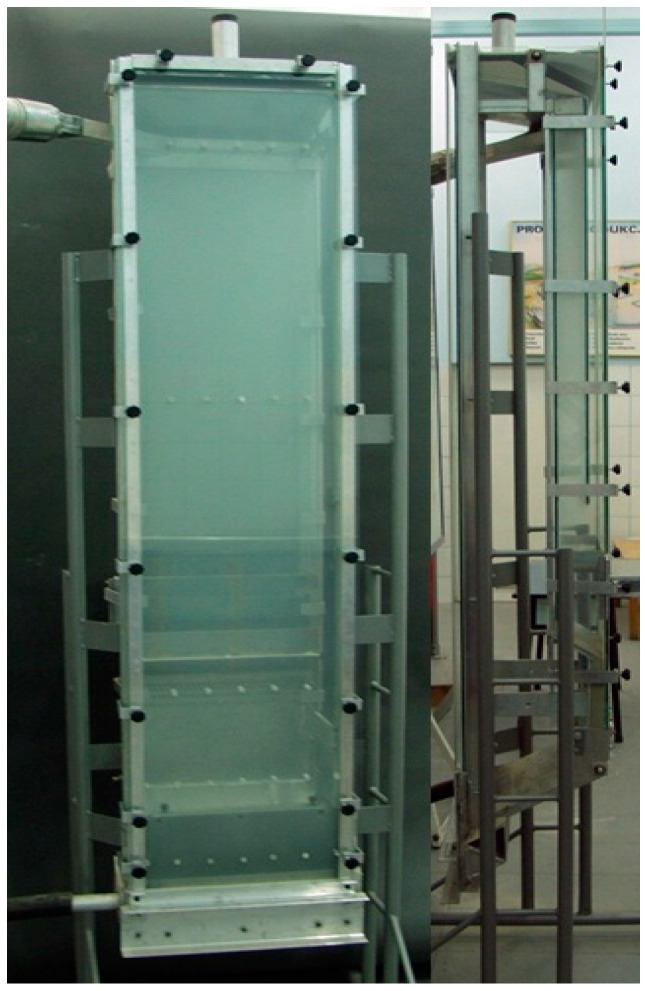
Fluidizing column: front view and side view.

**Figure 4:**
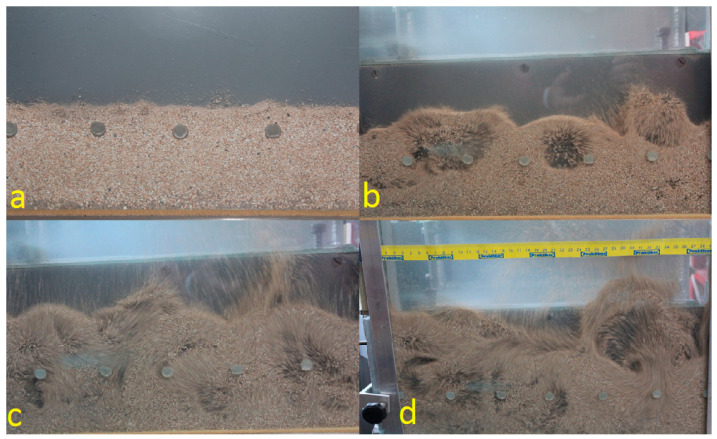
Development of a fluidized layer made of calcined material with increasing fluidization velocity: (**a**) filtration, fluidization velocity Uf < 0.4 m/s, (**b**) start of bubble fluidization, Uf = 0.4 m/s, (**c**) fluidization bubble fluidization, 0.4 < Uf < 0.9 m/s, (**d**) developed bubble fluidization, Uf > 0.9 m/s.

The experimental tests verifying the preliminary calculations were carried out on the stand present in the Department of Thermal Machinery—Figure 2 and Figure 3. In the first stage, tests on the fluidization process of material A, with a granule size <2 mm, with a mass of 2000 g were carried out after roasting at a temperature of 850 °C. In the second part of the research, 720 g of raw material A with a granule size <2 mm was sprinkled into the column. In both cases, the granular material was well fluidized. The transition from filtration to bubble fluidization was gentle. The material’s tendency towards channelling and fountaining was not noticed. The addition of the raw material did not change the fluidization process. The fluidization process was carried out at ambient temperature, i.e., 20 °C. It was noticed that, during the operation, the finest particles of the material were blown out of the fluidized column. It is to be expected that the finest fractions of the material will be blown out of the fluidized bed reactor, and an additional separation system should be used for the blown material, e.g., in the form of bag filters. During the test, it was noticed that the bubble fluidization process started at a fluidization velocity Umf = Uf = 0.4 m/s—Figure 4. This velocity is twice as high as that calculated theoretically. This is a result of the large amount of very fine material in the bed, which reduces the equivalent diameter calculated on the basis of the entire population of particles.

## 4. Preliminary Tests of the Calcination Process of Raw Clay Material at a Temperature of 850 °C in a Fluidized Bed

**Figure 5:**
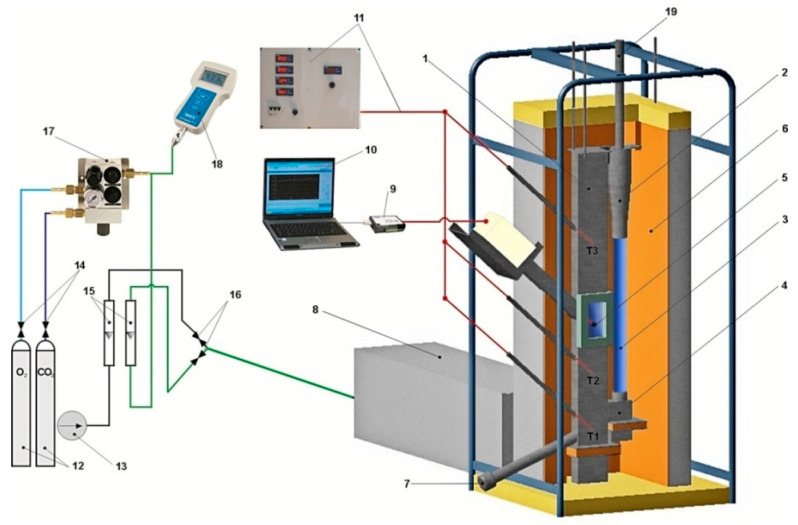
Diagram of a stand with a circulating fluidized bed for combusting solid fuels: 1—fluidization column, 2—cyclone, 3—downpipe, 4—return system, 5—combustion chamber, 6—insulation, 7—drain pipe, 8—air heater, 9—measurement card, 10—computer, 11—measurement system and temperature control, 12—cylinders with technical gases, 13—compressor, 14—pressure reducers, 15—rotameters, 16—control valves, 17—gas mixer, 18—oxygen analyser, 19—exhaust extractor.

The tests for the clay calcination process were carried out on a laboratory stand with a power of 12 kW, as presented in Figure 5. The stand, with a circulating fluidized bed, consisted of a fluidization column (1), a cyclone (2), a downpipe (3) and a return system (4). The main element of the stand was a flat fluidization column (1) with dimensions of 680 mm × 75 mm × 35 mm. The column is encased in segments of heaters, then covered with thermal insulation (6) and a metal cover. The fluidizing medium was air, which was supplied from the compressor (13). The air volume flow was measured using a rotameter (15). An air heater (8) was placed in front of the combustion chamber to ensure the proper temperature in the combustion chamber. The temperature control system (11) was based on four LUMEL microprocessor controllers, operating independently in the combustion chamber and in the air heater. The temperature in the combustion chamber was measured at three levels (T1–T3) using Pt-Rh10-Pt thermocouples, while in the air heater, it was measured using NiCr-NiAl thermocouples.

**Figure 6:**
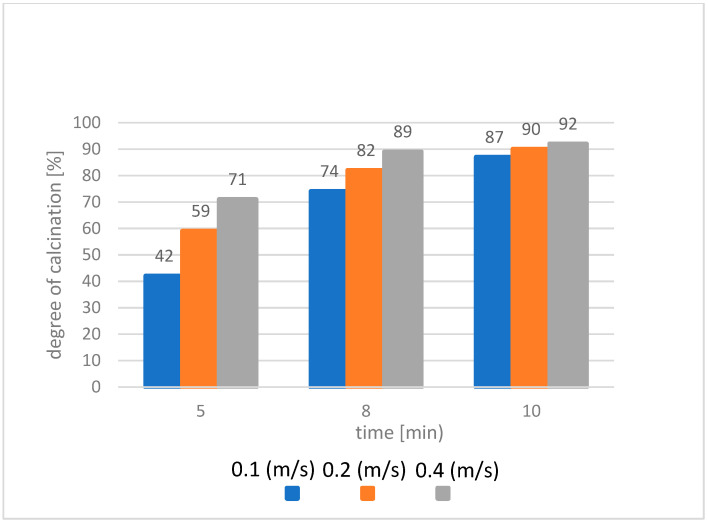
The dependence of the degree of calcination on the time and velocity of fluidization.

After the fluidized bed reached a temperature of 850 °C, a sedimentary rock clay material with a mass of 120 g (with a volume corresponding to the 300 g of quartz sand used in this stand) was introduced into the chamber as a substitute for the fluidizing material. Clay material has a much lower bulk density than quartz sand (approximately 590 g/cm^3^). The tests were performed for different velocities of the flowing fluidizing medium: 0.4; 0.2 and 0.1 m/s. The tests were carried out for three different residence times of the sedimentary rock in the fluidization chamber: 5, 8 and 10 min. During the tests, it was possible to sense the characteristic, irritating and unpleasant smell of the gaseous compounds emitted. At the lowest flow velocity, approximately 0.1 m/s, the circulating fluidized bed phenomenon could not be perceived in the fluidization chamber. This probably indicates that the flow was too low and that there may have been an underdeveloped bubble fluid layer (unfortunately impossible to observe). Moreover, it was noticed that the smallest and lightest fractions of sedimentary rock were expelled through the exhaust chimney immediately after being fed into the chamber. The results of the tests performed to determine the degree of calcination, presented in Figure 6, led to the conclusion that in order to obtain a high degree of calcination, the residence time of the raw clay material in the fluidized bed should be 10 min.

## 5. Experimental Studies of Fluidized Bed Calcination in a Fluidized Bed Reactor

After determining the fluidization parameters and conducting preliminary experimental studies, the following materials were calcined in the fluidized bed reactor:

- at a temperature of 850 °C: A, granule size < 2 mm; C, granule size < 2 mm; B, granule size < 5 mm; D, granule size 1–4 mm;
- at a temperature of 750 °C: B, granule size < 3 mm; TJW, granule size 1–3 mm;
- at 700 °C: B, granule size < 1 mm;

The mass of the sample of material D, with a granule size < 1 mm, after conducting the analysis and preliminary tests, turned out to be insufficient for the needs of the fluidized calcination experiment.

### Experimental Stand

**Figure 7:**
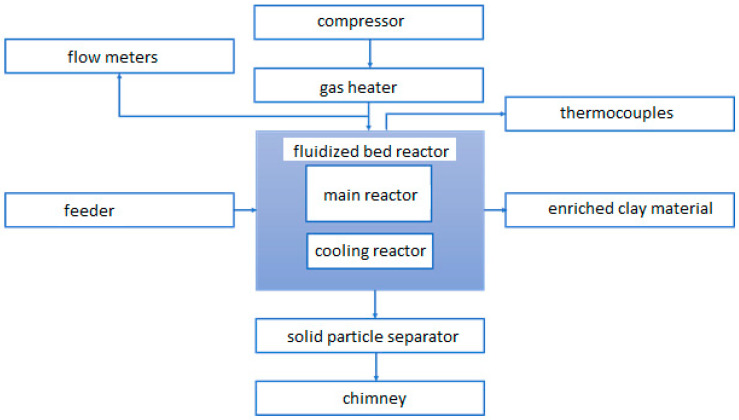
Block diagram of the stand for fluidized calcination of clay material.

The main element of the test stand was a fluidized bed reactor. A block diagram of the test stand is shown in Figure 7. The stand was additionally equipped with a cyclone for the dedusting of exhaust gases and the material that is being subjected to calcination. The rate at which material was fed into the reactor ranged from 1 to 3 g/s. The temperature was measured by means of thermocouples in three areas, successively: the main reactor chamber, the cooling reactor chamber, and the outlet. The reactor chamber was covered segmentally by four heaters, 1.5 KW each, in order to heat it up and maintain the set temperature in the range of 650–850 °C. The start-up of the reactor took about 125 min. The air, which was the fluidizing medium, was supplied by the compressor, then sent to the heater, where, after being heated to the set temperature in the range of 650–850 °C, it was transported through the reactor grate. The velocity of the fluidizing medium was controlled by a valve system. It should be noted that it was possible to control the gas flow rate independently in both chambers of the reactor. Regulation was carried out within a range of 0–11 m^3^/h in the first chamber and 0–5 m^3^/h in the second chamber. Control of the feeder, the power of the heaters and the set temperature was performed using a controller.

## 6. Results of BZ 1-103-3/2016/P Experimental Studies on Calcination in a Fluidized Bed Reactor

After calcination in the fluidized bed, the loss on ignition of the obtained calcinate was determined, and this was then compared with the loss on ignition for the raw materials, as presented in Table 5, and the degree of fluidized calcination was determined. In the case of material B, with a granule size of 5 mm, the fluidized calcination tests in the reactor were unsuccessful, because according to Geldart’s classification, this material belongs to group D, i.e., materials prone to fountaining. After the calcination process, the tested materials were re-analysed for particles and sent to the laboratory to determine the change in chemical composition after calcination and the degree of calcination. In the case of calcination at a temperature of 850 °C, the degree of calcination was nearly 100% in all of the analysed cases; for a temperature of 750 °C, the degree of calcination was 96.53% (material B, with a granule size of 1–3 mm) and 97.52% (material B, with a granule size < 3 mm). At a temperature of 700 °C, for material B with a granule size < 1 mm, the degree of calcination was 87.62%.

**Figure 8:**
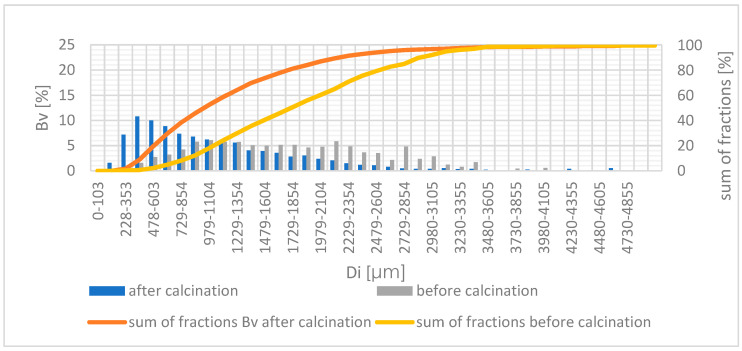
Volume distribution before and after the calcination process for the material D, with a granule size of 1–4 mm.

During the calcination process, the clay material was fragmented, as can be seen in Figure 8, which shows comparisons of BZ 1-103-3/2016/P particle size distribution before and after the calcination process.

The impact of the process of calcination on the chemical composition of the analysed clays was also analysed. On the basis of the analysis conducted, the chemical composition did not change under the influence of calcination. Oxides do not react to each other, nor do they transform under the influence of high temperatures.

## 7. Conclusions

1. The conducted analysis indicates that calcination of the clay granulate, which is highly aluminous in fluidized conditions, is possible while still preserving the appropriate granularity class with a range of between 0.5 and 3.0 mm. Fine-grained granulates above 0.5 mm are blown out with the exhaust fumes in the fluidization process, while granules over 3 mm do not create a constant fluid phase.
2. In the case of performing calcination at a temperature of 850 °C, very good calcination results were obtained when the granulate was held in the fluidized bed reactor for a 10-min period of time spent (the degree of calcination was then 100%).
3. It was also established that it is possible to calcine clay particles at lower temperatures, e.g., 750 °C, with a calcination degree of 96.5%, and at 700 °C, with a calcination degree of 87.6%.
4. During the time period in which the clay material particles remain in the fluidized bed reactor, they are subject to fragmentation, as is visible by the increase in the number of particles with smaller particle sizes in the graphs that show the particle distribution of materials after calcination.
5. Calcining in the fluidized bed does not change the chemical composition of the clay, as the oxides that make up the clay do not react to high temperatures, and the carbon contained in it is completely burnt out.
6. The research conducted illustrates that the process of calcinations under the conditions described in this paper require a period of time that is several times shorter than when using a rotary furnace, thus shortening the process by tens of minutes.
7. Due to the possibility of reactor remaining in continuous operation, this technology can find application in industry (while maintaining the appropriate grain size distribution to be subjected to calcination).

## Figures and Tables

**Table 1:** The results of the chemical analysis of clay raw materials [23].

Component	A	B	C
SiO_2_ wt.%	54.10	58.50	58.90
Al_2_O_3_ wt.%	41.70	32.70	32.80
Fe_2_O_3_ wt.%	1.07	2.51	2.01
TiO_2_ wt.%	0.58	2.01	1.52
CaO wt.%	0.16	0.33	0.22
MgO wt.%	0.24	0.61	0.45
K_2_O wt.%	0.37	2.99	2.16
Na_2_O wt.%	0.05	0.11	0.05
other wt.%	1.73	0.24	1.89
total wt.%	100	100	100

**Table 2:** Results of technical analysis of clay raw materials.

Material	Granulation [mm]	Moisture [%]	Volatile Matter Content [%]	Clay Content [%]
A	<2	6.20	1.78	92.02
B	<5	8.54	1.15	98.63
	<1	0.12	1.01	98.87
	<3	0.08	1.06	98.45
	1–3	0.11	0.61	97.43
C	<2	7.00	2.05	90.95
D	1–4	2.44	19.42	78.14
	<1	0.93	18.36	80.71

**Table 3:** Results of particle analysis of clay raw materials.

Material	Granulation [mm]	Mass [g]	Number of Particles [–]	Time [min]	*d_n_* ^1^ [μm]	*d_s_* ^2^ [μm]	*d_v_* ^3^ [μm]	*d_a_* ^4^ [μm]	*d_geo_* ^5^ [μm]	*D_med_* ^6^ [μm]	*D_mod_* ^7^ [μm]
A	<2	6.7	545,435	96	164.7	224.7	291	492	102.5	134.6	24.2
B	<5	14.5	151,073	18.3	57.5	200	434.3	2059.3	33	26.6	24.2
	<1	12	1,300,381	157.7	177	226.7	265.7	366	113.7	174	24.2
	<3	14.5	882,081	102.7	159.7	219.7	288.3	496.3	97.2	119.1	24.2
	1–3	13.3	443,342	162	242.7	179.7	288	1311	40.2	24.9	24.2
C	<2	10.6	385,442	32.7	80.2	193.7	343.7	1103	40.1	24.9	24.2
D	1–4	8.7	22,676	22	165.3	353.3	579.3	1363	74.1	43.4	51.6
	<1	10.1	146,958	28.7	155.7	199.3	242.7	360.3	115.3	113.7	51.6

^1^ $d_{n}$—arithmetic-mean particle diameter, μm. ^2^ $d_{s}$—surface mean diameter, μm. ^3^ $d_{v}$—volume mean diameter, μm. ^4^ $d_{a}$—Sauter mean diameter, μm. ^5^ $d_{geo}$—geometric diameter, μm. ^6^ $D_{med}$—median, μm. ^7^ $D_{mod}$—mode, μm.

**Table 4:** Results of calculations of fluidization parameters.

Material	Granulation (mm)	$\mathrm{Material}\;\mathrm{Density}\;(\rho_{m})\;(\mathrm{kg}/m^{3})$	$d_{a}(m)$	Ar (–)		Re (–)		U_mf_ (m/s)		U_t_ (m/s)		U_tr_ (m/s)		U_mb_ (m/s)	
T (°C)				27	850	27	850	27	850	27	850	27	850	27	850
A	<2	2500	0.000492	10,003	458	6.68	0.34	0.22	0.10	5.23	0.45	5.43	0.81	0.05	0.03
B	<5	650	0.002059	190,453	8724	65.05	5.90	0.50	0.41	8.89	0.76	5.40	0.81	0.20	0.14
	<1	2500	0.000366	4118	188	2.93	0.14	0.13	0.05	3.89	0.33	4.75	0.71	0.04	0.02
	<3	2500	0.000496	10,267	470	6.84	0.35	0.22	0.10	5.28	0.45	5.45	0.81	0.05	0.03
	1–3	650	0.001311	49,140	2251	25.19	1.64	0.30	0.18	5.67	0.48	4.40	0.66	0.13	0.09
C	<2	650	0.001103	29,265	1341	16.78	0.99	0.24	0.13	4.77	0.41	4.07	0.61	0.11	0.07
D	1–4	650	0.001363	55,223	2530	27.51	1.84	0.32	0.19	5.89	0.50	4.48	0.67	0.13	0.09
	<1	2500	0.0003603	3928.46	179.78	2.80	0.13	0.12	0.05	3.83	0.33	4.72	0.70	0.04	0.02

**Table 5:** Loss on ignition and degree of calcination of the examined clay materials.

Material	Granulation (mm)	Raw State	State after Fluidized Calcination	Degree of Calcination—State after Fluid Calcination	Fluidization Temperature	Fluidization Velocity
A	<2	12.85%	0.00%	100.00%	850 °C	0.12 m/s
B	<5	10.10% *	0.00% *	100.00% *	850 °C	0.45 m/s
	<1	10.10%	1.25%	87.62%	700 °C	0.06 m/s
	<3	10.10%	0.25%	97.52%	750 °C	0.11 m/s
	1–3	10.10%	0.35%	96.53%	750 °C	0.19 m/s
C	<2	14.15%	0.00%	100.00%	850 °C	0.15 m/s
D	1–4	26.00%	0.03%	99.88%	850 °C	0.21 m/s

* the degree of calcination was calculated on the basis of the recovered material after fluidization failure.

## Data Availability

The data presented in this study are available on request from the corresponding author.

## Supplementary Materials

The following are available online at https://www.mdpi.com/article/10.3390/ma14143989/s1, Figure S1: Quantitative, volumetric and area distribution, A < 2 mm, Figure S2: Quantitative, volumetric and area distribution, B < 5 mm, Figure S3: Quantitative, volumetric and area distribution, B < 3 mm, Figure S4: Quantitative, volumetric and area distribution, B < 1 mm, Figure S5: Quantitative, volumetric and area distribution, B 1–3 mm, Figure S6: Quantitative, volumetric and area distribution, C < 2 mm, Figure S7: Quantitative, volumetric and area distribution, D 1–4 mm, Figure S8: Quantitative, volumetric and area distribution, D < 1 mm.
