# Supplementary material for: Calcination of Clay Raw Materials in a Fluidized Bed
Source: Materials (Basel). 2021 Jul 16;14(14):3989. doi: 10.3390/ma14143989 (PMC8303566; doi:10.3390/ma14143989)
Supplement: Supplementary file 1 [file materials-14-03989-s001.zip › materials-1238454-SI.pdf]

materials

Supplementary Material

Calcination of Clay Raw Materials in A Fluidized Bed

Katarzyna Kaczyńska *, Konrad Kaczyński and Piotr Pełka

Czestochowa University of Technology, Faculty of Mechanical Engineering and Computer Science, Institute of Thermal Machinery, al. Armii Krajowej 21, 42-201 Czestochowa, Poland; konrad.kaczynski@pcz.pl (K.K.), piotr.pelka@pcz.pl (P.P.)
* Correspondence: katarzyna.kaczynska@pcz.pl

1. Results of particle analysis of clay raw materials

Figures 1-8 present the average quantitative, volumetric and surface distribution (minimum of three measurements) of all the samples. The curved line marked in red colour presents the sum of the fraction. Samples A-C were analysed in different measurement ranges on the AWK equipment than the samples of D.

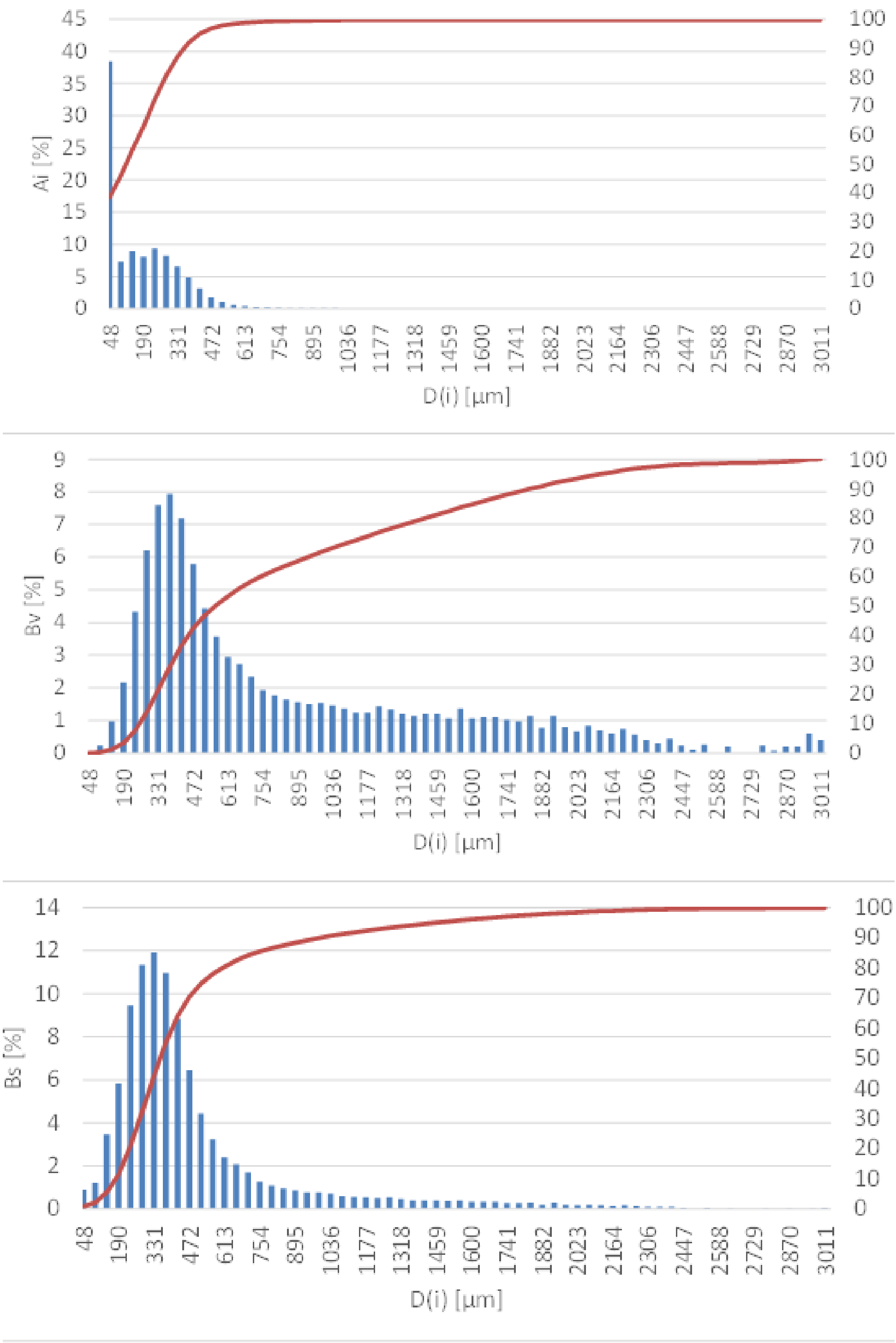

Figure S1. Quantitative, volumetric and area distribution, A <2mm.

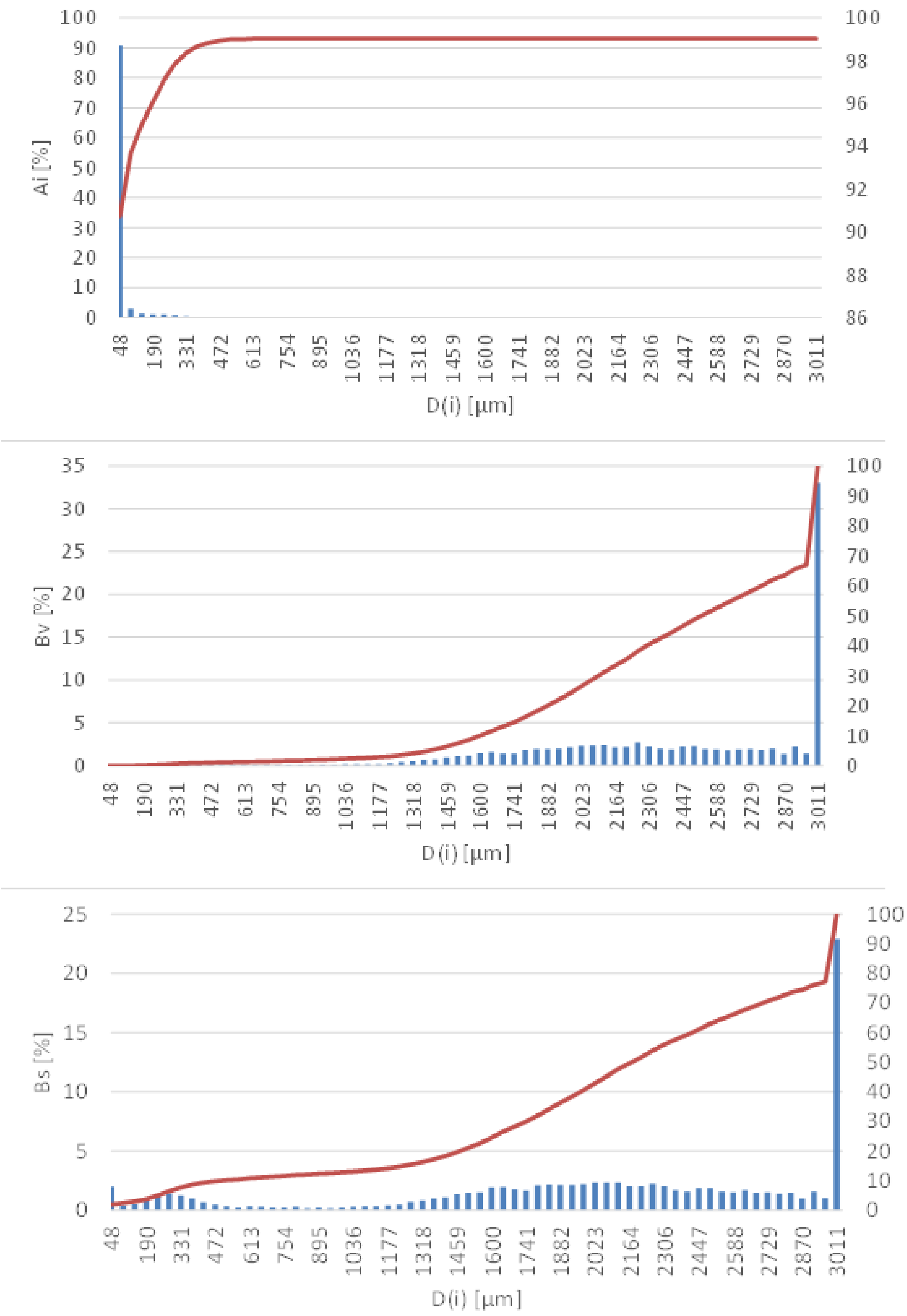

Figure S2. Quantitative, volumetric and area distribution, B <5mm.

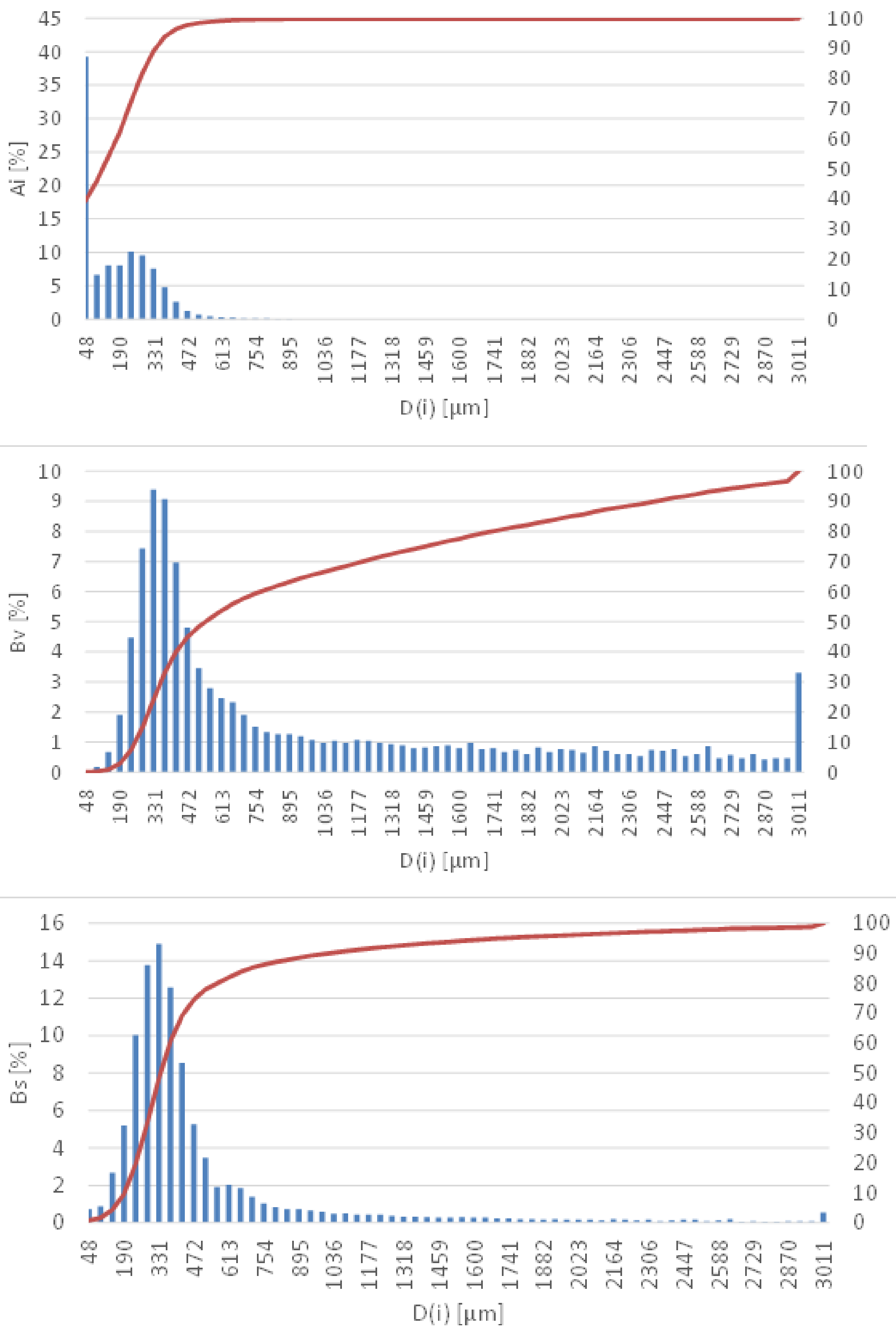

Figure S3. Quantitative, volumetric and area distribution, B <3mm.

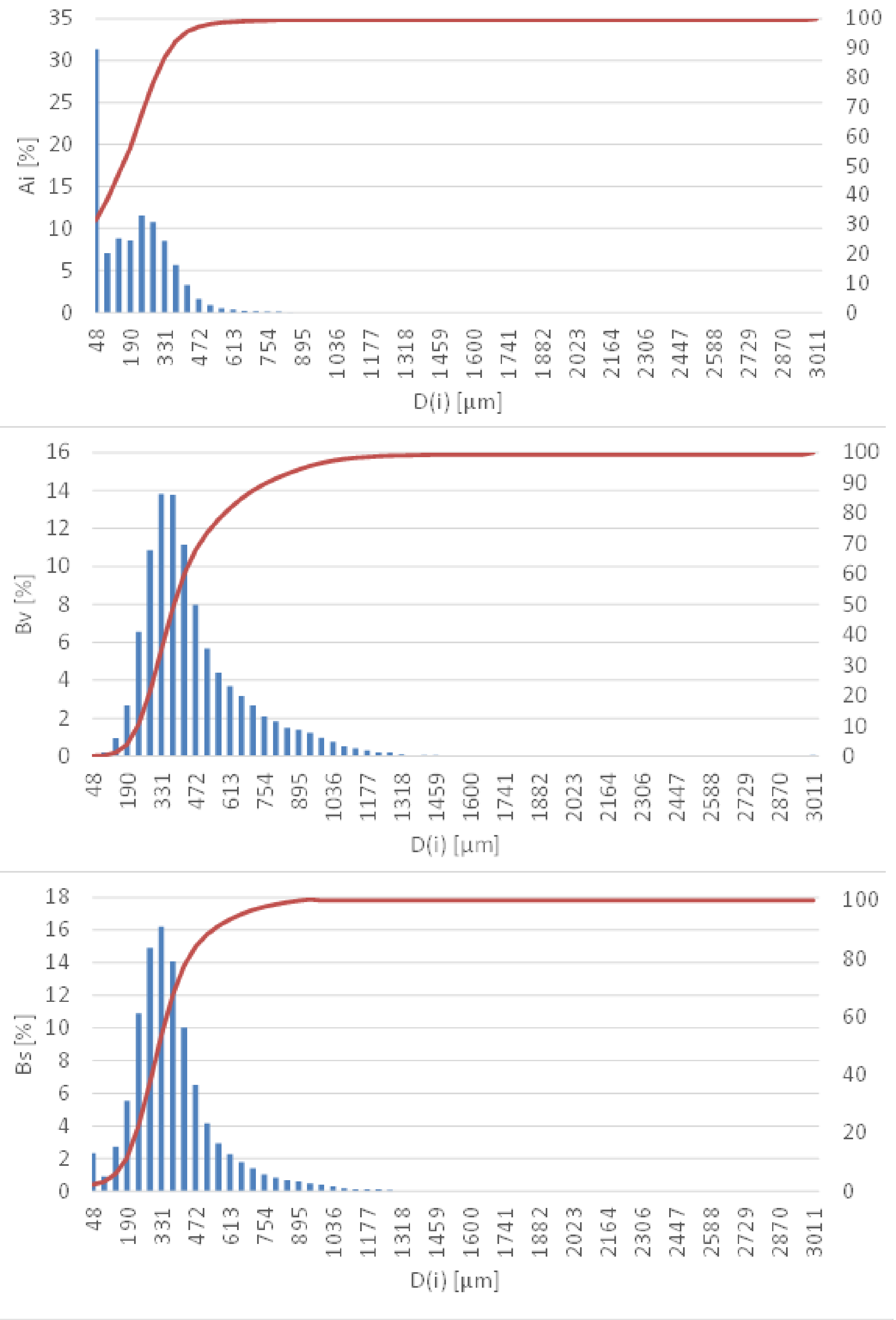

Figure S4. Quantitative, volumetric and area distribution, B <1mm.

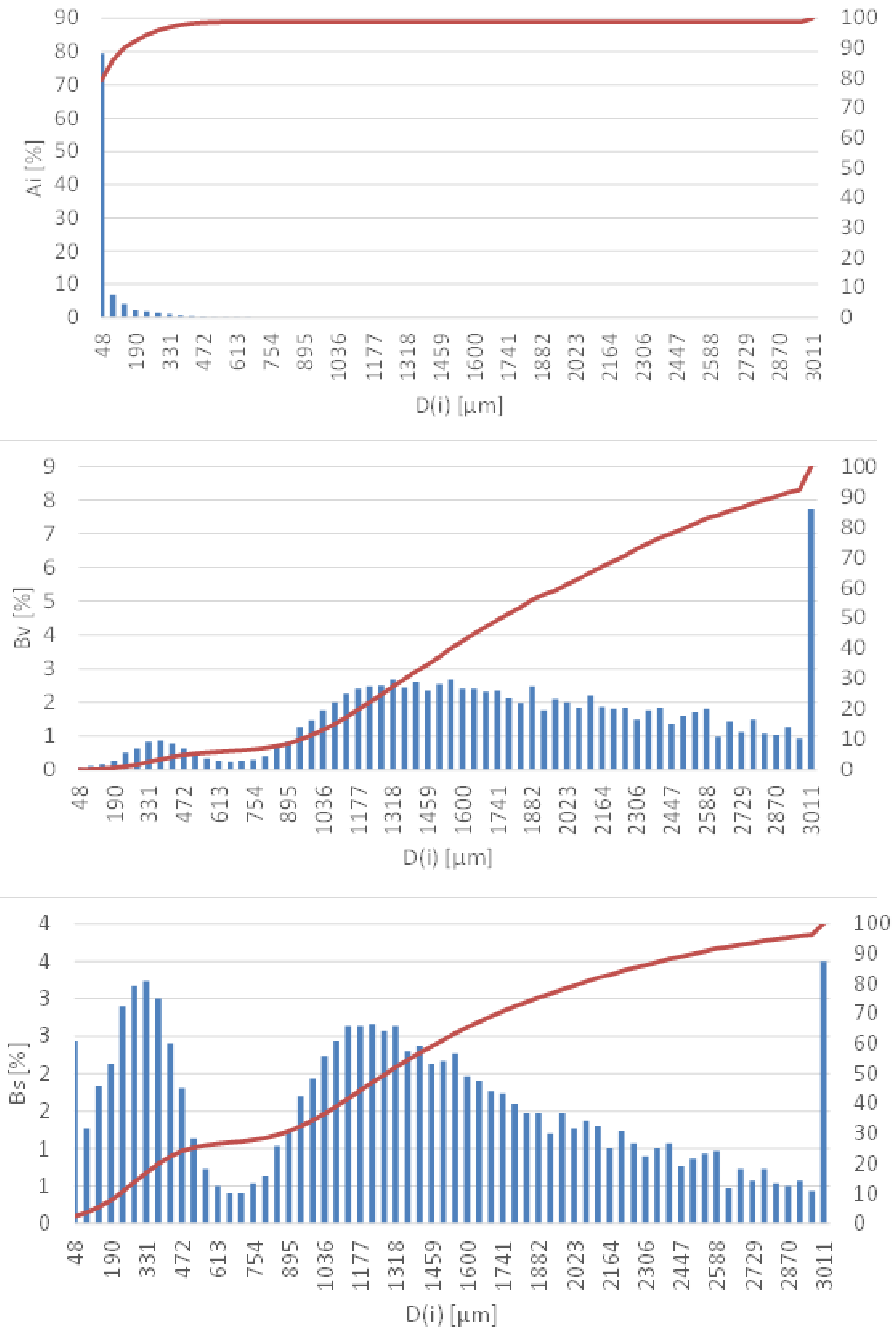

Figure S5. Quantitative, volumetric and area distribution, B 1-3mm.

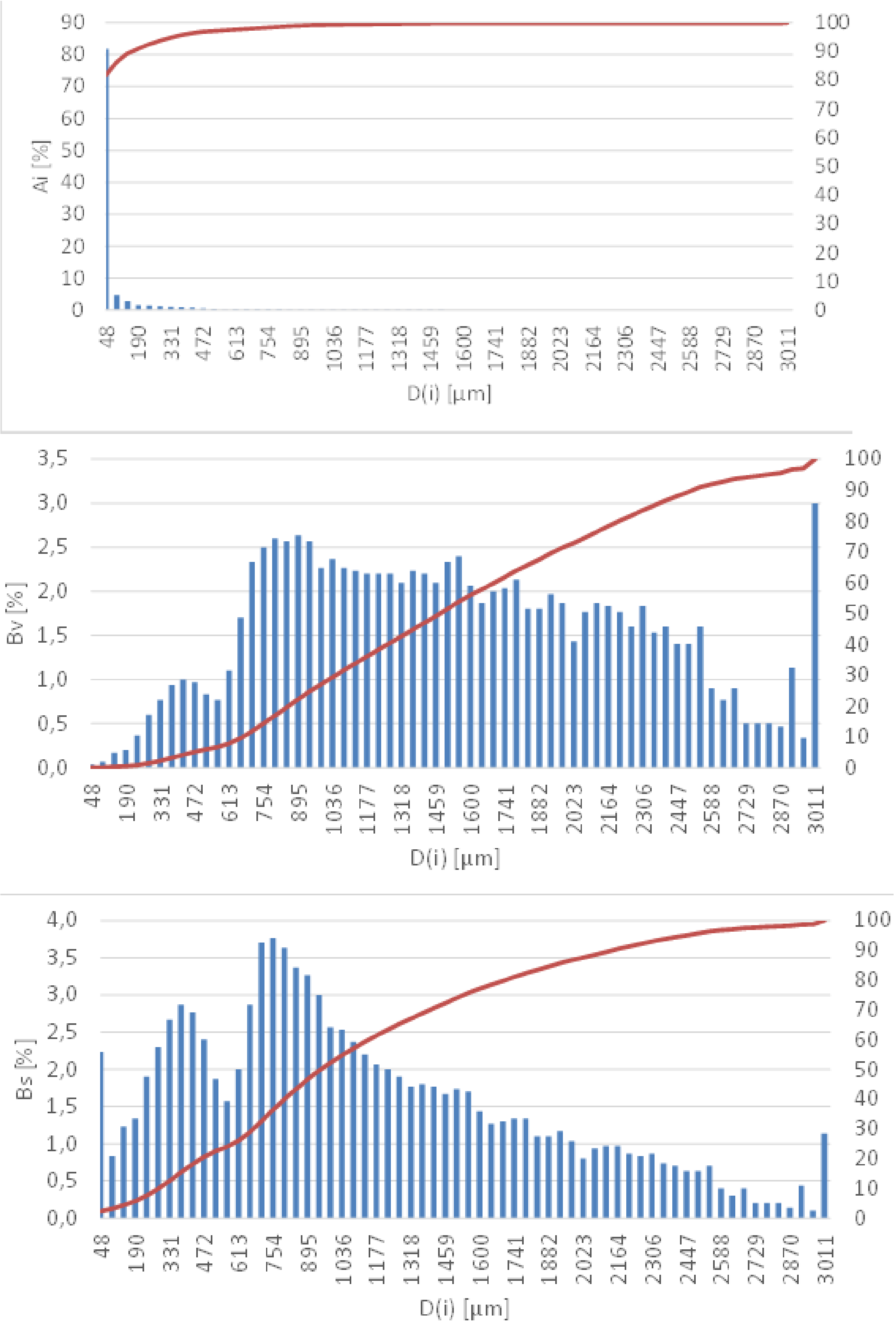

Figure S6. Quantitative, volumetric and area distribution, C <2mm.

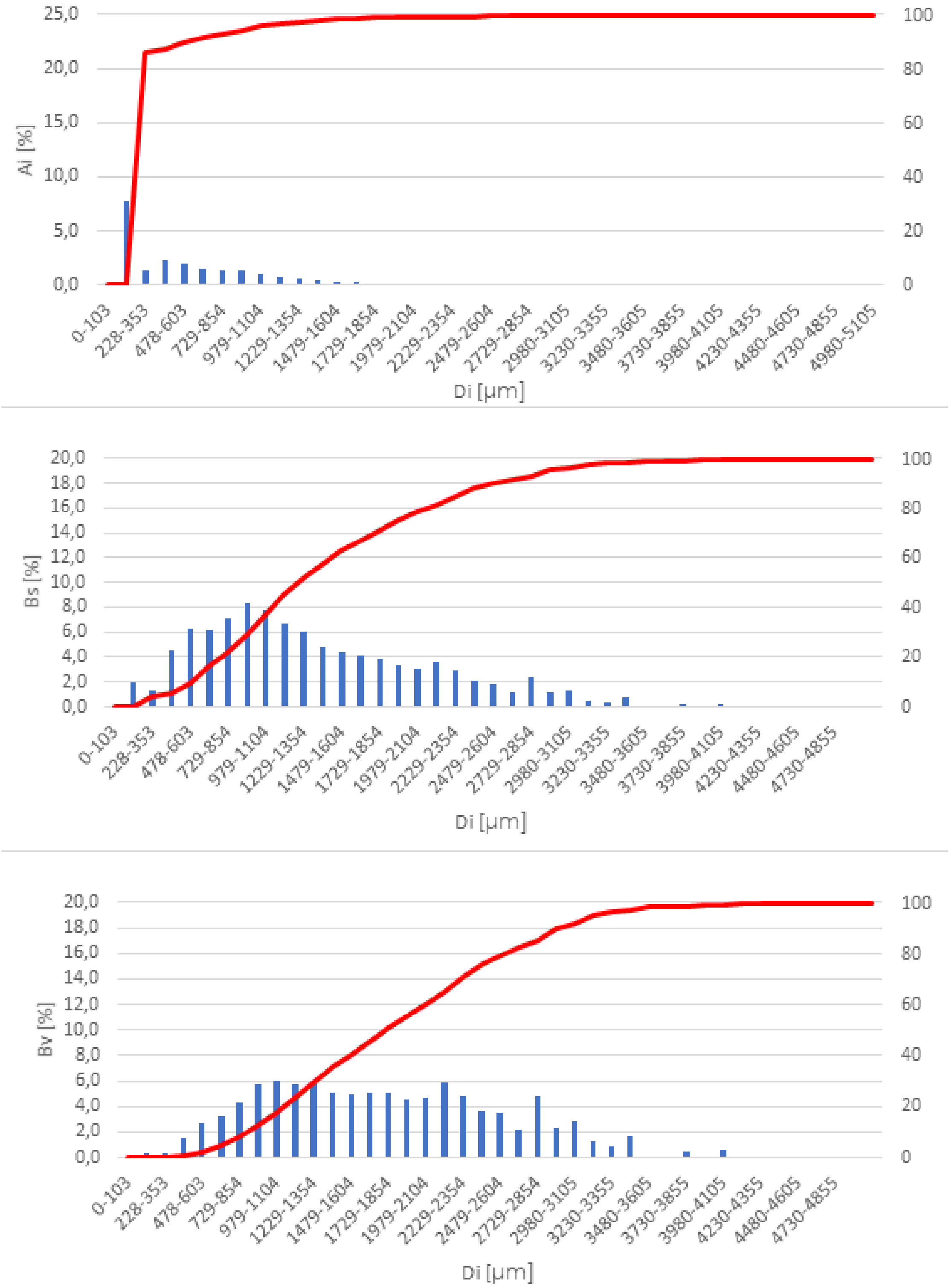

Figure S7. Quantitative, volumetric and area distribution, D 1-4mm.

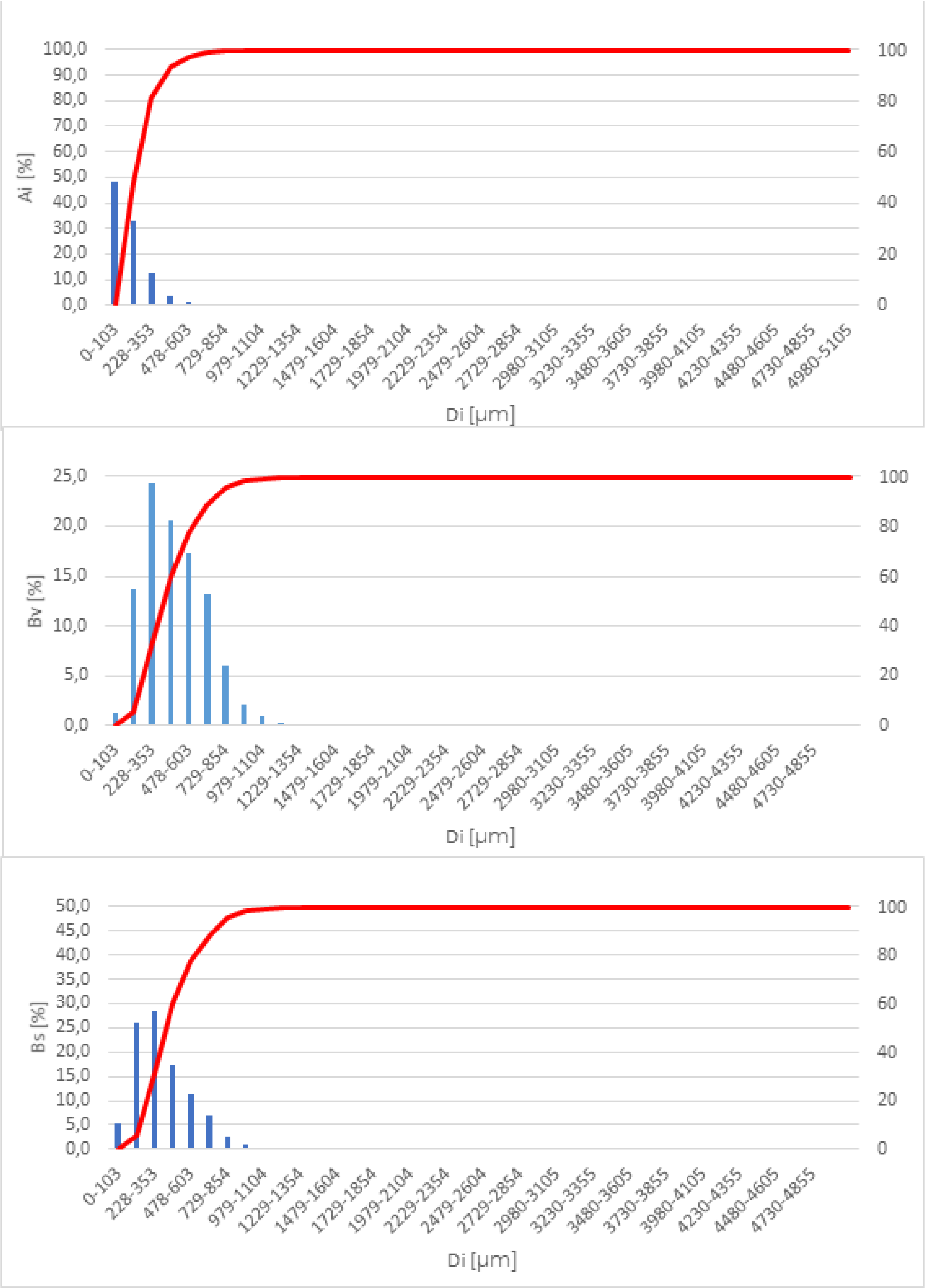

Figure S8. Quantitative, volumetric and area distribution, D <1mm.
